# Uncommon *MET* mutational landscape in a non-small cell lung cancer patient treated with crizotinib: Case report

**DOI:** 10.1016/j.heliyon.2024.e31944

**Published:** 2024-05-27

**Authors:** Margaux Geier, Jessica Nguyen, Estelle Dhamelincourt, Hélène Babey, Renaud Descourt, Gilles Quéré, Gilles Robinet, François Lucia, Mathilde Pacault

**Affiliations:** a Department of Medical Oncology, CHRU Morvan, University Hospital of Brest, Brest, France; b Radiation Oncology Department, University Hospital, Brest, France; c LaTIM, INSERM, UMR 1101, University of Brest, ISBAM, UBO, UBL, Brest, France; d Department of Medical and Molecular Genetics, CHRU Morvan, University Hospital of Brest, Brest, France

**Keywords:** Case report, Non-small cell lung cancer, *MET*, Resistance mechanisms, Crizotinib

## Abstract

**Background:**

*MET* exon 14 (*MET*ex14) skipping mutations are oncogenic drivers observed in approximately 3–4% of non-small cell lung cancers (NSCLC). Several distinct genetic alterations leading to METex14 have been reported but clinical significances of rare mutations are not well defined as well as outcomes of patients upon MET inhibitors (METi).

**Case presentation:**

This report presents the case of a patient with metastatic NSCLC harboring an uncommon *MET* mutational landscape including notably a novel *MET*ex14 mutation (R1022L). Dramatic but transient efficacy was observed under crizotinib, due to early occurrence of acquired both on- and off-target mechanisms of resistance such as MET D1246H mutation and wild-type *KRAS* amplification.

**Conclusion:**

Our case provides additional data on *MET* rare oncogenic variants and their sensitivity to METi. Systematic assessment of post-tyrosine kinase inhibitor tumor sample remains critical to identify on- and off-target mechanisms that may represent therapeutically targetable drivers in resistant patients.

## Highlights

• Resistance to MET inhibitors inevitably occurs in *MET* exon 14-dependent NSCLC.
• Acquired on and off-target resistance mechanisms are not fully elucidated.
• Data on *MET* rare oncogenic variants are lacking, especially their sensitivity to MET inhibitors.
• Additional data on R1022L *MET* new variant are provided in this report.
• D1246H second-site mutation and wild-type *KRAS* amplification emerged as resistance mechanisms to crizotinib.

## 1 Background

*MET* encodes for a tyrosine kinase receptor of hepatocyte growth factor that activates RAS/ERK/MAPK and PI3K/AKT intracellular signaling pathways. Permanent activation of MET receptor may lead to oncogenesis. *MET* exon 14 (*MET*ex14) skipping mutations are oncogenic drivers observed in approximately 3–4% of non-small cell lung cancers (NSCLC) and up to 20–30 % of sarcomatoid carcinomas. These alterations preferentially occur in older patients with smoking history and are associated with poor prognosis. They rarely co-exist with other known driver mutations [1].

Several distinct genetic alterations leading to *MET*ex14 have been reported but clinical significances of rare mutations are not well defined as well as outcomes of patients upon MET inhibitors (METi).

Here we report the case of a patient with metastatic NSCLC harboring an uncommon *MET* mutational landscape. Dramatic but transient efficacy was observed under type I METi crizotinib, due to early occurrence of acquired both on- and off-target mechanisms of resistance.

## 2 Case report

**Fig. 1:**
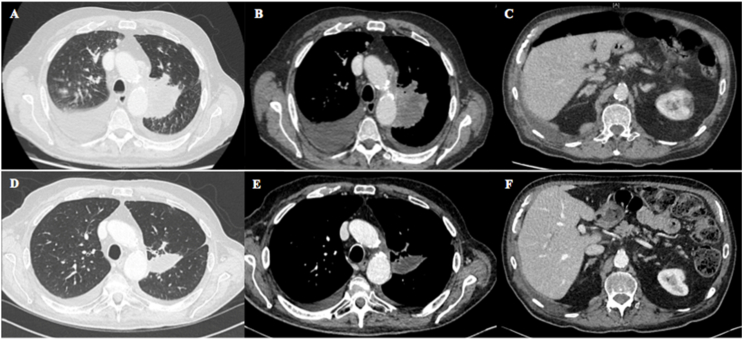
**A-C:** Baseline CT scan; **D-F:** CT scan performed 2 months after crizotinib initiation.

**Fig. 2:**
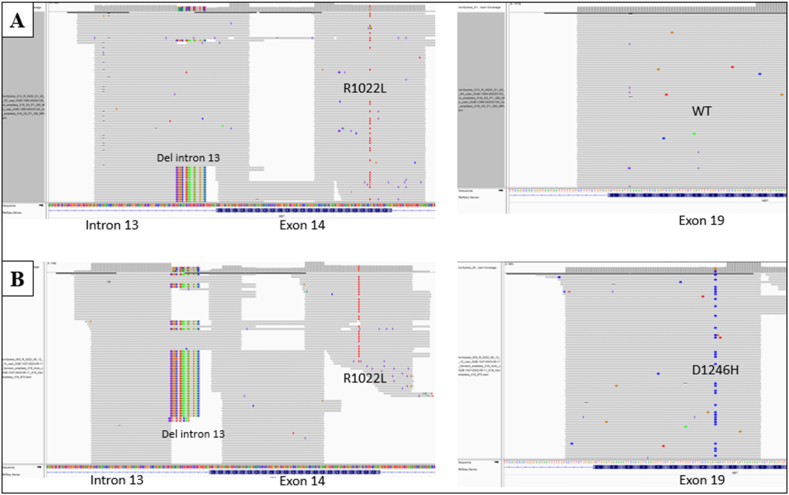
*MET* mutational landscape pre **(A)** and post **(B)** crizotinib, identified by DNA sequencing. WT: Wild-type.

A 79-year-old white man presented to Hospital Emergency Department in January 2022 with acute respiratory failure. He was a former smoker with a 25-pack-year smoking history and quit thirty years ago. He reported occupational asbestos exposure until age 50. The contrast CT scan showed a lung tumor mass located in the left upper lobe with contralateral pulmonary lesions, pleural and pericardial effusions, liver, adrenal, bone and muscular metastases (Fig. 1A–C). The patient underwent bronchial biopsy via flexible bronchoscopy and pleural aspiration. A diagnosis of stage IV PD-L1 ≥50 % lung adenocarcinoma was subsequently made. Critical immunohistochemistry (IHC) biomarkers (*ALK*, *ROS1*, *NTRK*, *RET*, *NRG1*) were negative. Molecular testing on the tumor detected a *MET* intron 13 mutation (c.2942-31_2942-1del) associated with a novel *MET*ex14 mutation (R1022L, c.3065G > T; p.(Arg1022Leu)) (Table 1, Fig. 2A). RNA-based assays confirmed *MET*ex14 skipping. No other co-mutation was noted.

**Table 1:** Tumor biological landscape before and after crizotinib.

Before crizotinib	After crizotinib
***MET* intron 13 mutation c.2942**–**31_2942-1del**	*MET* intron 13 mutation c.2942-31_2942-1del
***MET*ex14 skipping mutation** **R1022L, c.3065G > T; p.(Arg1022Leu)**	*MET*ex14 skipping mutation R1022L, c.3065G > T; p.(Arg1022Leu)
	*MET* exon 19 mutation D1246H, c.3736G > C; p.(Asp1246His)
**Wild-Type *KRAS***	Wild-Type *KRAS* amplification
**PD-L1** ≥ **50 %**	PD-L1 = 90 %

Because of a poor performance status, patient was not eligible to first-line immunotherapy or cytotoxic chemotherapy. Palliative radiotherapy of painful bone metastases was initiated. Despite clinical deterioration, crizotinib was administered orally at a starting dose of 250 mg twice daily in continuous 28-day cycle. After 2 weeks of therapy, we observed clinical and radiographical evidences of tumor response. METi was well tolerated and patient only reported low-grade peripheral edema of uncertain etiology. Partial objective tumor response was observed after 8 weeks of crizotinib (Fig. 1D–F).

After 12 weeks, patient experienced novel episode of acute respiratory failure due to bilateral pleural effusion. Therapeutic pleural aspiration was performed to relieve symptoms. METi-related pleural toxicity was ruled out by cytological testing that confirmed malignant effusion. Comprehensive genomic profiling highlighted heterogeneous mutational landscape with previous original *MET* intron 13 and *MET*ex14 mutations in addition to acquired *MET* exon 19 mutation (D1246H, c.3736G > C; p.(Asp1246His)) (Fig. 2B) found in the COSMIC database (COSM689) and wild-type *KRAS* amplification. To note, strong PD-L1 expression (90 %) was observed (Table 1). Unfortunately, CT scan showed thoracic progression. Crizotinib was therefore discontinued but patient was not eligible to type II METi nor chemo/immunotherapy and received best supportive care. Due to cancer progression, he died two weeks after, i.e. 5 months after cancer diagnosis.

## 3 Discussion

*MET*ex14 skipping mutations are rare actionable genetic events in NSCLC. METi such as crizotinib, capmatinib, tepotinib have demonstrated efficacy in advanced NSCLC harboring such alterations and represent a dramatic therapeutic option for patients not eligible to first line chemo/immunotherapy, especially for older patient and/or not enough ”fit” people [2]. Furthermore, METi have been shown to be generally well tolerated, notably crizotinib, that remains useful in certain circumstances [3].

Comprehensive genomic profiling on solid tumor samples or analyses of circulating tumor DNA on noninvasive liquid biopsies is particularly important to identify oncogene-addicted NSCLC but also inevitable polyclonal resistance. To our knowledge, the R1022L *MET*ex14 variant has not been previously reported and clinical actionability of this rare alteration is not elucidated. This mutation present here at diagnosis is a single nucleotide missense variant of uncertain clinical significance in solid cancers according to ClinVar interpretation. However, a synergistic effect between the deletion within intron 13 of *MET* and this novel *MET*ex14 variant cannot be ruled out, but it is quite probable that the presence of R1022L mutation alone might not be incriminated in exon 14 skipping. Indeed, deletion within intron 13 upstream might already disrupt splice acceptor site.

The lack of prolonged response to crizotinib in our patient was explained by early occurrence of heterogeneous acquired resistance mutations including *MET*-dependent and *MET*-independent resistance mechanisms. Acquired *MET* p.(D1246H) exon 19 mutation has already been described in 2 patients with *EGFR*-mutant, *MET*-amplified NSCLC after progression on osimertinib combined either with savolitinib (4) or crizotinib [5]. In the first case, patient also displayed on-target and off-target resistance mechanisms [4]. In addition, this activating *MET* mutation in exon 19 was also observed after progression during the sequential therapy with alectinib and crizotinib in a metastatic NSCLC patient harboring primary *ALK* rearrangement and secondary acquired *MET*ex14 mutation [6]. Bahcall et al. reported acquired amplification of wild-type *KRAS* as a parallel signaling behind crizotinib resistance using *MET*ex14-mutant patient-derived cell line and xenografts [7] and proposed therapeutic approach of concomitant inhibition of MET and PI3K. Recently, amplification of the MAPK pathway effector genes was recurrently found at resistance to METi in a serie of 20 patients [8].

Interestingly, strong PD-L1 expression (90 %) was observed at progression in pleural effusion. Indeed, MET triggered a transcriptional increase of PD-L1, promoting tumor immunoescape [9,10]. Whether PD-L1 overexpression is part of underlying mechanisms of crizotinib resistance in our case, remains to be further investigated.

## 4 Conclusion

Outcomes of a patient with rare potential oncogenic variant are valuable to guide treatment decisions, although with a low level of evidence. Acquired wild-type *KRAS* amplification and *MET* secondary mutations may emerge as preponderant mechanisms of resistance for both common and uncommon *MET*ex14 skipping mutations targeted with METi. Systematic assessment of post-tyrosine kinase inhibitor tumor sample remains critical to identify on- and off-target mechanisms that may represent therapeutically targetable drivers in resistant patients. Nonetheless, whether patients might benefit from a combination treatment approach deserves further investigations.

## Ethics statement

Written informed consent was obtained from the individual for the publication of any potentially identifiable images or data included in this article.

## Data availability statement

Data will be made available on request.

## CRediT authorship contribution statement

**Margaux Geier:** Writing – review & editing, Writing – original draft, Validation, Supervision, Methodology, Data curation, Conceptualization. **Jessica Nguyen:** Project administration, Methodology, Data curation, Conceptualization. **Estelle Dhamelincourt:** Methodology, Investigation, Conceptualization. **Hélène Babey:** Data curation, Conceptualization. **Renaud Descourt:** Visualization, Validation, Supervision, Methodology, Data curation, Conceptualization. **Gilles Quéré:** Validation, Supervision, Data curation, Conceptualization. **Gilles Robinet:** Validation, Supervision, Conceptualization. **François Lucia:** Supervision, Methodology, Formal analysis. **Mathilde Pacault:** Writing – original draft, Visualization, Validation, Supervision, Methodology, Formal analysis, Data curation, Conceptualization.

## Declaration of competing interest

The authors declare that they have no known competing financial interests or personal relationships that could have appeared to influence the work reported in this paper.
